# The Role of Virtual Consultations in Plastic Surgery During COVID-19 Lockdown

**DOI:** 10.1007/s00266-020-01932-7

**Published:** 2020-08-31

**Authors:** Vikram Sinha, Mohsan Malik, Nora Nugent, Paul Drake, Naveen Cavale

**Affiliations:** 1 School of Medical Education, King’s College London, Great Maze Pond, London, SE1 1UL UK; 2 Department of Ophthalmology, Moorfields Eye Hospital, London, UK; 3 Plastic Surgery, Queen Victoria Hospital, East Grinstead, UK; 4 Surgical Department, King’s College Hospital, London, UK

**Keywords:** Plastic surgery, Telemedicine, Virtual consultation, Patient safety, Technology

## Abstract

**Background:**

COVID-19 has led to government enforced ‘lockdown’ in the UK severely limiting face-to-face patient interaction. Virtual consultations present a means for continued patient access to health care. Our aim was to evaluate the use of virtual consultations (VCons) during lockdown and their possible role in the future.

**Methods:**

An anonymous survey was disseminated to UK and European plastic surgeons via social media, email sharing and via the European Association of Societies of Aesthetic Plastic Surgery newsletter. Uptake of VCons, modality, effectiveness, safety and future utility were assessed.

**Results:**

Forty-three senior plastic surgeons responded to the survey. The majority of the respondents (97.7%) reported using VCons during COVID-19 lockdown, of which 74.4% had no prior experience. Two-thirds of surgeons utilised commercial platforms such as Zoom, FaceTime and Skype, 38.1% of respondents did not know about or were unsure about adequate encryption for health care use, and just under a half (47.6%) reported they were unaware of or lacking GDPR compliance. Most (97.6%) say they are likely to use virtual consultations after lockdown.

**Conclusion:**

Virtual consultations have had a crucial role in patient care during UK lockdown. It is clear that they will serve as an adjunct to face-to-face consultation in the future. Further regulation is required to ensure platforms offer adequate safety and security measures and are compliant with relevant data protection laws.

**Level of Evidence IV:**

This journal requires that authors assign a level of evidence to each article. For a full description of these Evidence-Based Medicine ratings, please refer to the Table of Contents or the online Instructions to Authors www.springer.com/00266.

**Electronic supplementary material:**

The online version of this article (10.1007/s00266-020-01932-7) contains supplementary material, which is available to authorized users.

## Introduction

The rapid spread of novel coronavirus (COVID-19), first identified in Wuhan province in December 2019, has led to a global pandemic and unprecedented challenge for health care institutions across the world [1]. In an attempt to contain the spread of COVID-19, the UK government implemented national curfew (‘lockdown’) policy on the 23 March 2020, to minimise community transmission. This has resulted in reduction of clinical activity across specialties demanding great flexibility from clinicians to reduce patient footfall whilst still prioritising emergencies. Face-to-face consultations were significantly reduced and outside of urgent or trauma settings and in many areas of elective practice, placed on hold entirely in an attempt to reduce the spread of disease between doctor and patient, resulting in significant restrictions to outpatient care [1]. Similar to allied medical specialties, plastic surgeons have made use of virtual consultations (VCons) to provide continued access to medical care whilst maintaining the rules of government lockdown [2]. VCons have been used to consult new patients and to reduce anticipated strain upon resumption of normal services by allowing surgeons to manage patients remotely [3].

VCons are a form of telemedicine, a term used to define any medical activity involving an element of distance. Telemedicine has been widely used in specialties such as general practice allowing patients to have a consultation with a doctor or nurse via telephone call without the need to see a clinician physically [4]. Refinement of existing video communication technology and a surge in broadband speeds mean that video communications are now more accessible, allowing patients to be examined virtually in some clinical situations. This has expanded the role of VCons into a potential tool to assess and consent patients pre-procedure and to follow-up and discharge patients where necessary [3]. The efficiency and cost-effectiveness of VCons have been demonstrated in UK-based randomised control trials [5]. Furthermore, the development of purpose-built apps for VCons such as Babylon^®^ has certified their use as secure and valid means of communicating with patients through their compliance with EU GDPR (General Data Protection Regulation) and HIPAA (Health Insurance Portability and Accountability Act) [6, 7].

Indeed, VCons have proven an effective tool for plastic surgeons during the COVID-19 lockdown. However, their role in future plastic surgery without or with reduced government-imposed social distancing measures is still evolving. The aim of this study was to evaluate the safety and utility of VCons during the COVID-19 lockdown, to consider its use in future practice, and, to provide guidance on safely and securely utilising and selecting a platform for VCons.

## Methods

### Survey

The survey consisted of an anonymous Google Forms (supplementary) and was disseminated to UK and European plastic surgeons. Face validity was performed by senior authors (NN, PD, NC), and further construct validity was performed on the first 10 responses to assess quality of data capture.

### Dissemination

The survey was disseminated via social media, email sharing and via the EASAPS (European Association of Societies of Aesthetic Plastic Surgery) newsletter. The survey collected responses for 2 weeks. Data were gathered and analysed using Google Forms, Google Drive and Google Sheets.

## Results

**Fig. 1:**
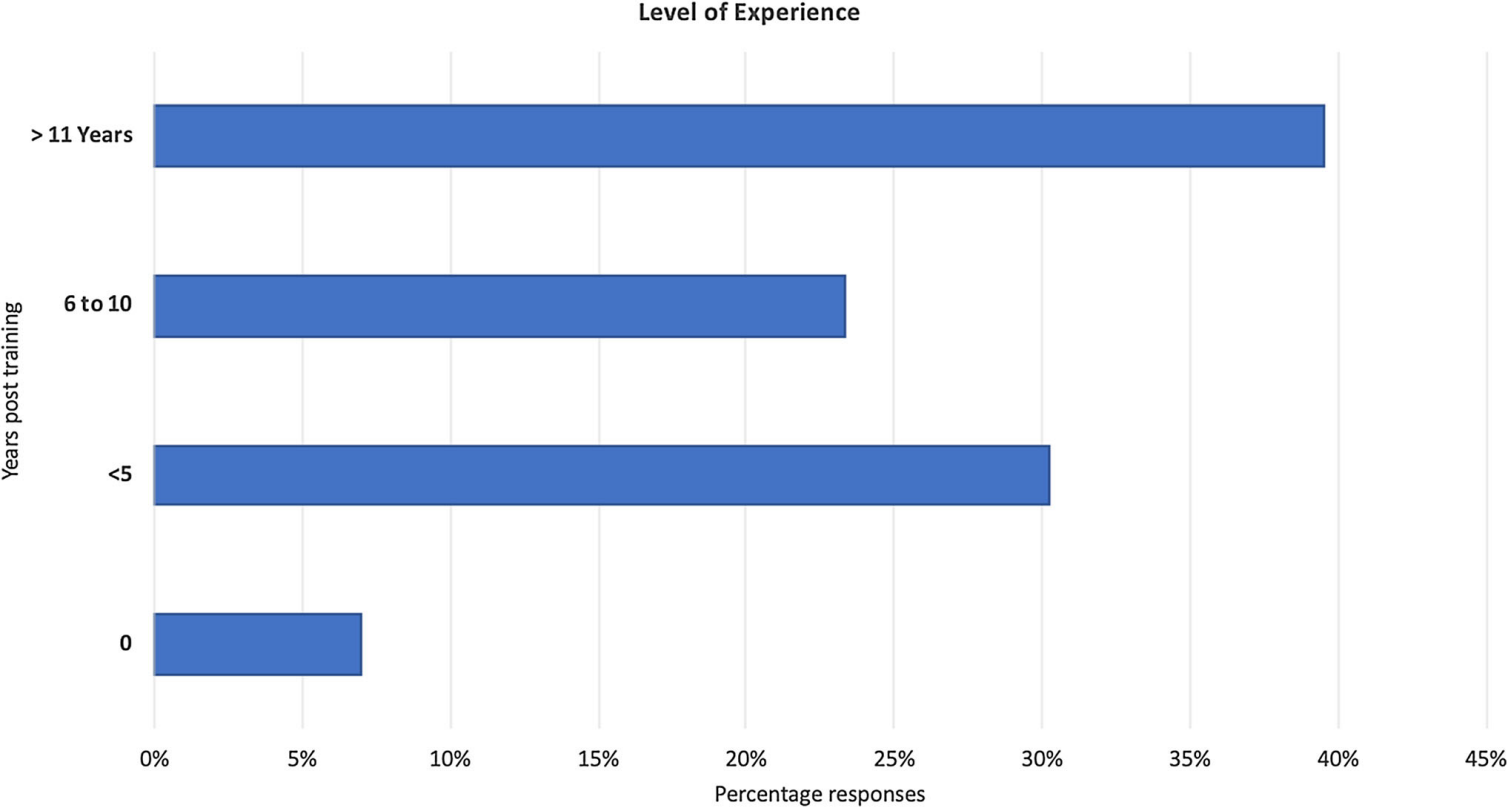
Range of reported experience amongst survey participants

*Demographics* 43 plastic surgeons responded to the survey, with mixed level of experience (Fig. 1), with aesthetic (69.8%), breast (58.1%) and skin cancer (44.2%) subspecialties most represented. The vast majority of the respondents (97.7%) reported using virtual consultations during COVID-19 lockdown, of which 74.4% had no prior experience.

**Fig. 2:**
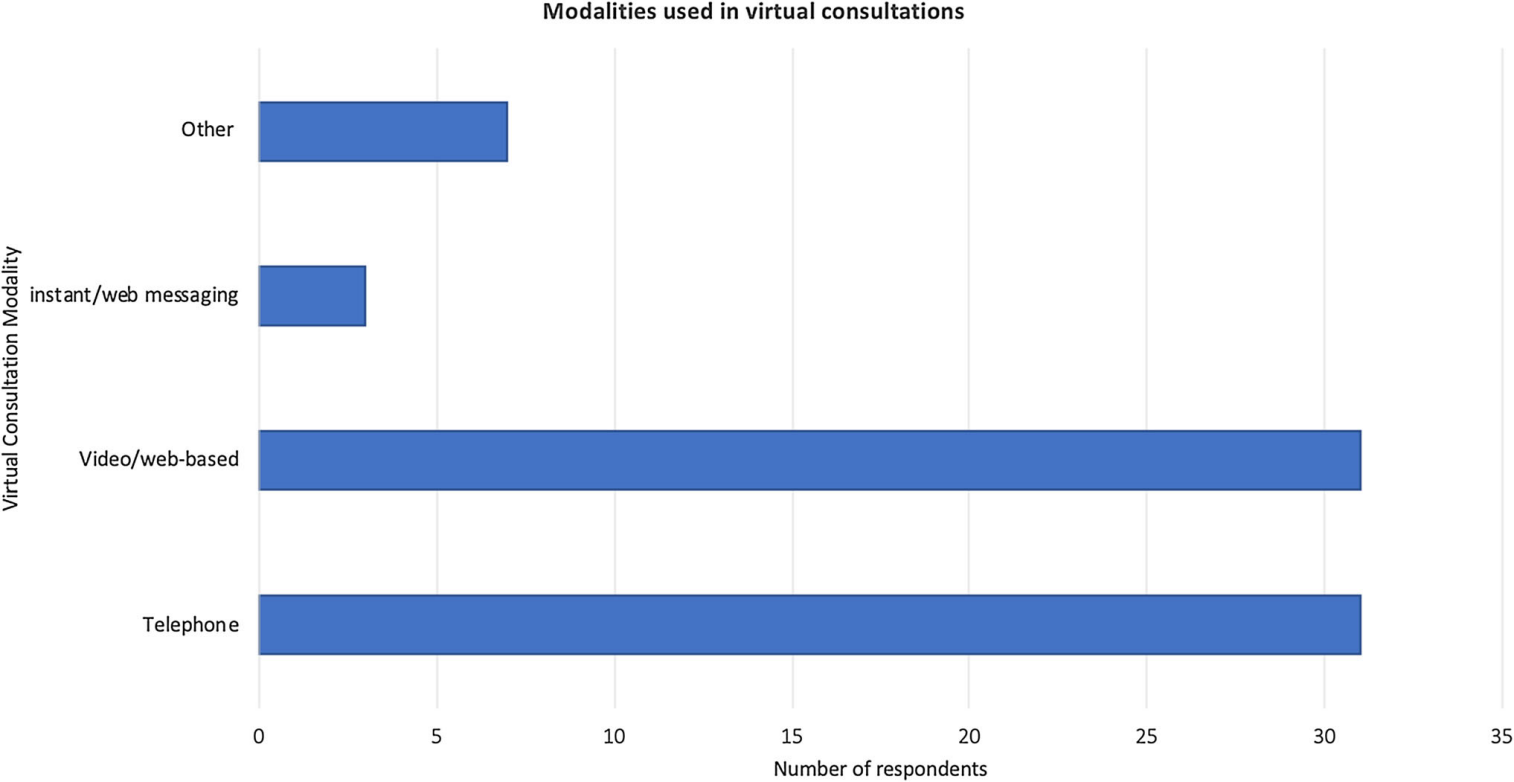
Virtual consultations modalities used amongst survey participants

**Fig. 3:**
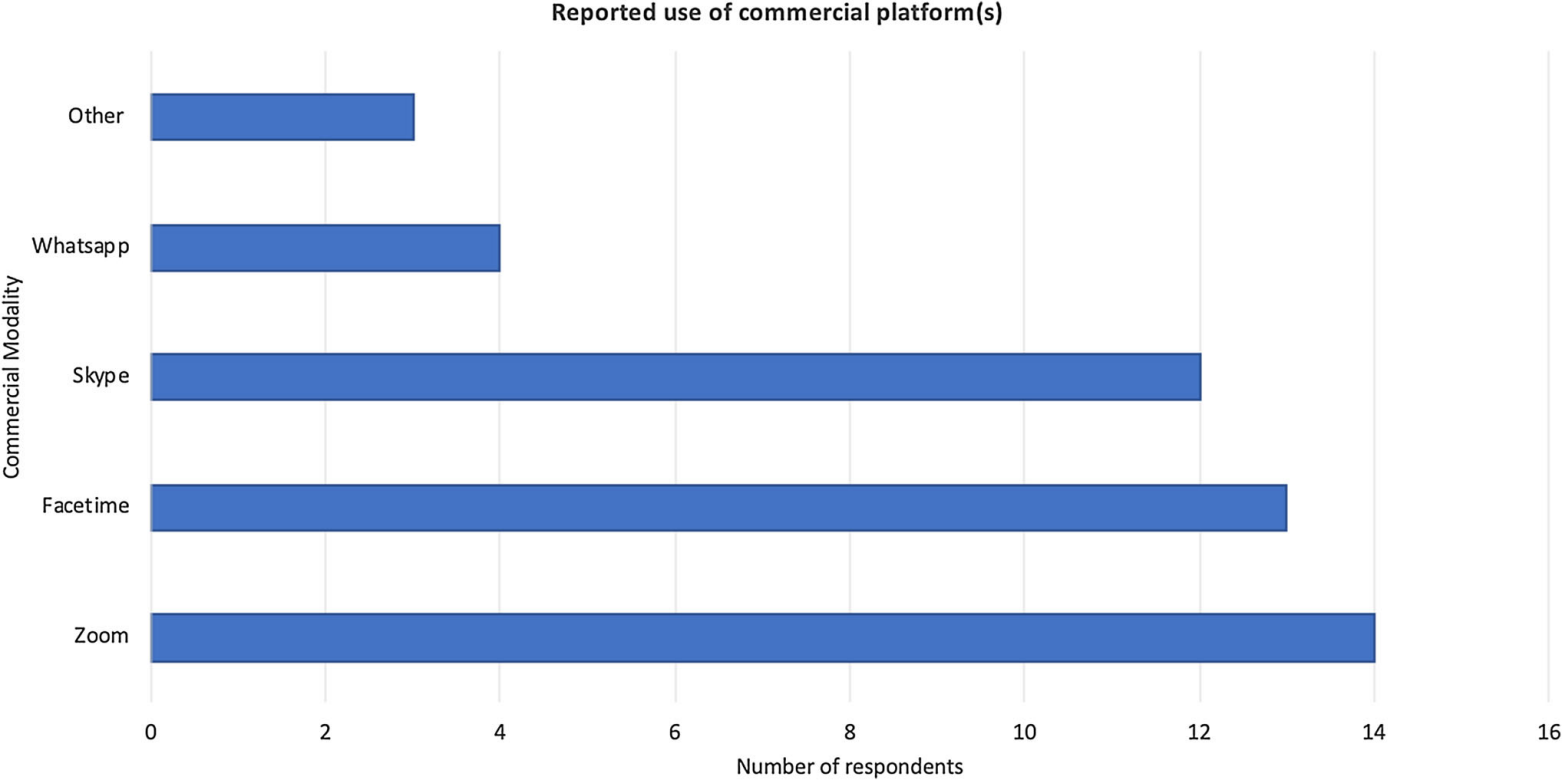
Reported use of commercial platform amongst survey participants

*Modality* 72.1% of the respondents used combination of telephone and video modalities for VCons, whereas web messaging and photograph telemedicine were rarely used (Fig. 2). Only one-third of doctors used purpose-built platforms, the most common being Doxy.me. The remainder opted for common commercial platforms used such as Zoom, FaceTime and Skype (Fig. 3).

**Fig. 4:**
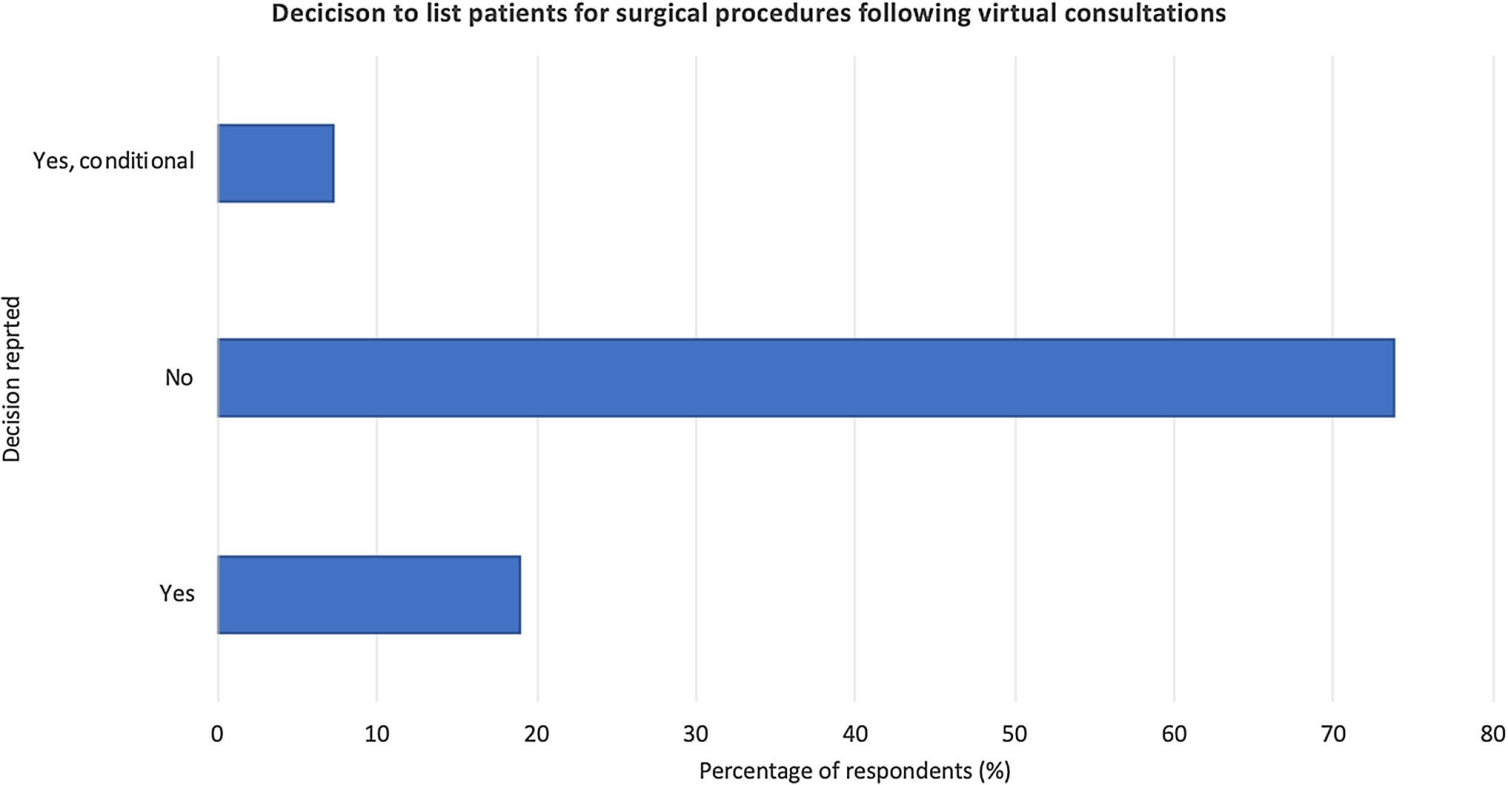
Percentage of respondents that reported listing patients for surgery after virtual consultations

*Consultation skills* Although 88.1% of doctors were confident with their virtual consultation skills, 50% claimed their communication skills were the same as face-to-face, and approximately a third (35.7%) stated their communication was better in face-to-face. The majority (69%) were not confident undertaking remote examinations and did not utilise virtual examination. Nineteen per cent of consultants felt they would list the patient for surgery following virtual consultation, in contrast to 54.8% who would prefer to review the patient for face-to-face consultation prior further management (Fig. 4).

**Fig. 5:**
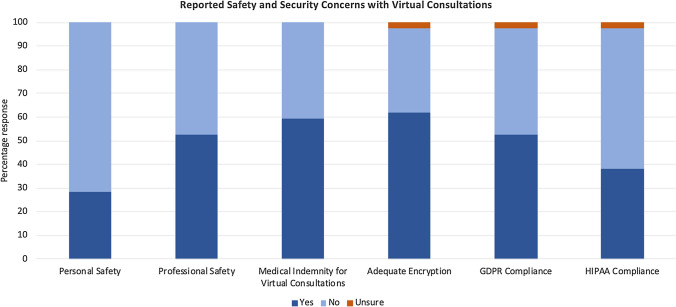
Reported safety and security concerns with virtual consultations

*Safety* 71.4% reported no concerns of personal safety during VCons. However, half reported concerns for professional safety and did not have or were unaware of medical indemnity for VCons. We found 38.1% of the respondents did not know about or were unsure about adequate encryption for health care use, and just under a half (47.6%) reported they were unaware of or lacking GDPR compliance (Fig. 5).

*Future utility* 97.6% say they are likely to use VCons after lockdown. 78% would prefer VCons to face-to-face in certain situations, such as an initial consultation or expression of interest. Those not using VCons stated that clinical concerns, safety and security online and legal concerns were the main reasons for not using VCons. Doctors stated peer or organisation pressure may convince them to start using VCons.

## Discussion

Our survey of plastic surgeons shows that the use of virtual consultations has been accelerated by COVID-19 lockdown with majority suggesting that they will continue to have a role alongside traditional face-to-face consultations in the future. However, as with any new technology, further guidance is required to ascertain the following: which platforms can be legally used, is communication sufficient to consent and discharge patients and which situations still require face-to-face consultations.

Only 32.7% of doctors were using purpose-built platforms, with the majority opting for commercial platforms such as Zoom, Skype and FaceTime. Purpose-built platforms ensure adequate encryption to prevent any unwanted intrusion of confidential information and are compliant with UK and EU digital information law [6, 8]. During the pandemic, emergency legislation and organisational guidelines were relaxed to allow platforms to be used to facilitate patient care. However, we anticipate future guidance to allow VCons over purpose-built platforms only to ensure safety and security of both doctor and patient. 41.5% of doctors in our survey were unaware of their indemnity status whilst utilising VCons. Currently, the UK General Medical Council (GMC) has not provided specific advice regarding indemnity cover for VCons. However, doctors are required to have adequate indemnity in place for all areas of their practice [9].

Adequate communication during VCons is essential for a plastic surgeon in order to inform and obtain adequate consent prior to later procedures. History-taking and interviewing patients is a key skill that surgeons develop at medical school and refine during their entire career; however, during VCons, verbal and non-verbal communication can be limited by technical and logistical constraints. Certain platforms can compress video making facial expressions difficult to read and hindering non-verbal communication, and in telephone consultations, non-verbal communication is lost entirely. Video-based consultations may also suffer from lag resulting in audio and video becoming unsynchronised [10]. However, other studies have shown that the presence of lag lead to better communication as the delay forced participants to consider their words with more thought and make greater use of turn-taking [11]. For procedures with fewer risks to communicate, VCons may be sufficient to consent and discharge; however, as our survey showed, the majority of surgeons (53.7%) would still prefer a face-to-face consultation before undertaking a procedure, particularly those which are more invasive. Based on our survey, we decided to formulate a checklist in order to aid clinicians in selecting a platform suitable for VCons (Table 1).

**Table 1:** Checklist of recommendations for virtual consultations

	Recommendation
System	Accessible and compatible with a variety of devices and operating systems
	Utilise minimal device storage and bandwidth
Environment	Quiet space with adequate lighting (or dedicated virtual consultations hub)
	Adequate privacy for exchange of confidential information
Interaction	Two-way exchange of information between patient and clinician
	Multi-modality consultations involving; text, audio and video
	Option for virtual chaperone or translator
Communication	Real-time communication
	Supplementary delayed communication (messages) for questions requiring further deliberation
	Video communication facility to allow non-verbal communication and clinical examination
Integration	Integration to patients’ medical records
	Integration with community/primary care
Regulatory compliance	Information Governance (GDPR and HIPAA compliant)
	Cyber essentials certification (CE and CE+)

Additional for VCons in NHS care

Integration of referrals to and from different NHS departments (e-consults)

NHS data Co-ordination Board standards: Clinical Risk Management (DCB 123 and 160)

Lengthier and more invasive procedures have more risks to communicate to the patient, and the surgeon has a duty when consenting to make sure the patient can understand, retain and weigh up the information given to them during consultation. Currently, there are no laws or guidelines describing comprehensively which procedures can be consented to virtually and which procedures require a patient to be seen in person. 58.5% of doctors we surveyed state that they believe VCons can be used instead of face-to-face for some treatments but not all; however, the decision is currently at the surgeon’s discretion. Examining a patient remotely can be problematic, 68.3% of our survey did not carry out remote examinations, and only 7.3% made constant use of chaperones. Some describe tasking their patients to self-assess pre-operation such as for lymphadenopathy in lieu of a conventional examination; however, lack of examiner skill may mask any significant pathology [12].

## Conclusion

Virtual consultations have proved to be both time and financially efficient and have allowed patients continued access to care during this period of social distancing. It is very likely that plastic surgeons will continue utilising VCons in the future as an adjunct to traditional face-to-face consultations. Currently, the laws are relaxed upon which platforms are permitted for health care use; however, we believe that GDPR and HIPAA compliant purpose-built platforms should be gold standard for future practice. VCons are currently not in a state to completely replace face-to-face for all procedures; however, guidance would be helpful in the future by our professional associations as VCons become more widely utilised and their advantages and limitations become more apparent.

## Supplementary Material

Copy of Google Forms survey questionnaire.

## Electronic supplementary material

Below is the link to the electronic supplementary material.

Supplementary material 1 (PDF 85 kb)
