# Supplementary material for: The Role of Virtual Consultations in Plastic Surgery During COVID-19 Lockdown
Source: Aesthetic Plast Surg. 2020 Aug 31;45(2):777–83. doi: 10.1007/s00266-020-01932-7 (PMC7458356; doi:10.1007/s00266-020-01932-7)
Supplement: Supplementary file 1 — Supplementary material 1 (PDF 85 kb) [file 266_2020_1932_MOESM1_ESM.pdf]

Virtual Consultations In Plastic Surgery

Survey of current and future practice

The responses are anonymised. Please enter email address to prevent duplication of responses.
*Required

1. Email address *

____

About you

2. How many years have you been practicing post-CCT? *

Mark only one oval.

- 0 (still training)
- <5 years
- 6-10 years
- > 11 years

3. Gender *

Mark only one oval.

- Female
- Male
- Prefer not to say
- Other: ______________________________

4. Subspeciality *

Tick all that apply.

- [ ] Aesthetic
- [ ] Breast
- [ ] Burns
- [ ] Congenital
- [ ] Craniofacial (including Occuloplastic, OMFS)
- [ ] Hand and Upper Limb
- [ ] Lower Limb Trauma
- [ ] Paediatric & Congenital
- [ ] Skin Cancer
- [ ] Microsurgery

Other: [ ] ____________________

Virtual Consultation

5. Have you conducted virtual consultations during COVID-19 lockdown? *

Mark only one oval.

- ( ) Yes
- ( ) No *Skip to question 38*

6. Were you hosting virtual consultations prior to COVID-19 Lockdown? *

Mark only one oval.

- ( ) Yes
- ( ) No
- ( ) NA

7. What is the format of your virtual consultation(s)? Please select all that apply *

Tick all that apply.

- [ ] Telephone
- [ ] Videocall/ web-based video consultation
- [ ] Instant/Web messaging

Other: [ ] ____________________

8. Have you used a purpose-built platform(s) for clinical consultations?

Mark only one oval.

( ) Yes
( ) No

9. If Yes, which application(s) have you used?

____

10. Do you use any of the following commercial platform(s) for clinical consultations? Please select all that apply

Tick all that apply.

- [ ] Zoom
- [ ] FaceTime
- [ ] Skype
- [ ] Google Hangout

Other: [ ] ____________________

11. How long is your average virtual consultation time? *

Mark only one oval.

- ◯ <5 minutes
- ◯ 5 - 15 minutes
- ◯ 15 - 30 minutes
- ◯ 30 - 60 minutes
- ◯ >60 minutes

12. Do you apply fees for virtual consultations? *

Mark only one oval.

- ◯ Yes - My fees are the same as face-to face appointment
- ◯ Yes - My fees are less than for face-to-face
- ◯ Yes - Usual public sector (NHS) Tariff
- ◯ Yes - Unsure on the public sector (NHS) Tariff
- ◯ No - I have waived the fees at present due to COVID-19
- ◯ No - I do not have fees for virtual consultations
- ◯ No - I do not have fees for any consultations
- ◯ Other: ____________________

13. Any comments to the questions in this section?

____

____

____

____

____

Virtual Consultations Utility

14. Are you confident with your remote consultation skills? *

Mark only one oval.

- Yes
- No

15. Do you think your communications skills during virtual consultations are *

Mark only one oval.

- Better than Face-to-Face Consultations
- Worse than Face-to-Face Consultations
- No Different to Face-to-Face Consultations
- Other: ______________

16. Are you confident undertaking remote examinations? *

Mark only one oval.

- Yes
- No

17. Do you give patients the option to refuse virtual examination?

Mark only one oval.

- Yes
- No
- Not Applicable
- Other: ______________

18. Do you use chaperones for virtual consultations? *

Mark only one oval.

- Always
- Sometimes
- Never
- Not Applicable

19. Do you list the patient for surgery or procedures following virtual consultations? *

Mark only one oval.

- Yes - after only a virtual consultation
- Yes - but I would also have a face to face consultation before treatment
- No
- Other: ______________________________

20. Do you discharge new patients following initial virtual consultations? *

Mark only one oval.

- Always
- Sometimes
- Never

21. Do you discharge follow-up patients following virtual consultations? *

Mark only one oval.

- Always
- Sometimes
- Never

22. If you have answered 'sometimes' to any of the above, please could you provide more details?

____
____
____
____
____

23. Which of the following best describes your opinion on the role of virtual consultations? *

Mark only one oval.

- Virtual consultations can be used instead of face to face consultations in all instances
- Virtual consultations can be used instead of face to face consultations for some treatments
- Virtual consultations should always be followed by a face to face consultation
- Virtual consultations should not be used in plastic surgery
- Other: ____________________

24. Any further comments on the role of virtual consultations?

____
____
____
____
____

Safety and Security Online

25. Do you have concerns about your personal safety online during virtual consultations? *

Mark only one oval.

- Yes
- No

26. Do you have concerns about your professional safety online during virtual consultations?

Mark only one oval.

- Yes
- No

27. Do you know or have medical indemnity for virtual consultations? *

Mark only one oval.

- Yes
- No

28. Does the virtual platforms you are using have adequate encryption for healthcare use? *

Mark only one oval.

- Yes
- No
- Not sure

29. Does the virtual platforms you are using is GDPR compliant? *

Mark only one oval.

- Yes
- No
- Not sure

30. Does the virtual platforms you are using is HIPAA compliant? *

Mark only one oval.

- Yes
- No
- Not Sure

31. Any comments to the questions in this section:

Future use of Virtual Consultations

32. Are you likely to use virtual consultations after COVID-19 lockdown? *

Mark only one oval.

- Yes
- No

33. When would you prefer to use virtual consultation over face to face? *

Mark only one oval.

- Always
- Sometimes
- Never

34. Please could you explain your answer to the question above *

35. When would you prefer to use face to face consultation over virtual? *

Mark only one oval.

- Always
- Sometimes
- Never

36. Please could you explain your answer to the question above *

37. How would you describe patient feedback towards virtual consultations *

Mark only one oval.

- Patients prefer virtual consultations
- Patients prefer face-to-face consultations
- Patients have no preference
- Other: ____________________

Skip to question 41

Not currently using virtual consultations

38. Which of the following reasons for not using virtual consultations apply to you? *

Tick all that apply.

- [ ] Clinical Concerns (please expand below)
- [ ] Security and Safety Concerns (please expand below)
- [ ] Fiscal Concerns (please expand below)
- [ ] Legal Concerns (please expand below)
- [ ] Ethical Concerns (please expand below)
- [ ] Poor confidence in virtual platforms
- [ ] Poor confidence in personal IT skills required
- [ ] Poor confidence in the IT technology
- [ ] Lack of access to appropriate equipment

Other: [ ] ____________________

39. Any comments on your answer above or reservations to virtual consultations?

40. What Factors would influence your likelihood of using virtual Consultations in the Future?

Tick all that apply.

- [ ] Resolutions of the reservations mentioned above
- [ ] Peer Pressure
- [ ] Fear of Missing Out
- [ ] Forced due to organisation/ institutional reasons

Other: [ ] ____________________

Thank you for participating in our survey

Please use the space below for any further comments you wish tc make.

41. Further Comments?

____

____

____

____

____

This content is neither created nor endorsed by Google.

Google Forms
